# Understanding Bleeding Risk after Anticoagulation

**DOI:** 10.1371/journal.pmed.0020352

**Published:** 2005-10-11

One class of treatments commonly used for prevention and treatment of venous and arterial thrombosis is vitamin K antagonists (VKA), such as warfarin. The molecular target of these anticoagulants is the vitamin K epoxide reductase complex (VKORC) of which one component, VKORC1, was recently identified. The gene for this component is mutated in individuals with combined deficiency of vitamin K–dependent clotting factors type 2 or with warfarin resistance.

What makes VKA treatment so tedious for patients is that the dose of VKA required to achieve anticoagulation varies among patients and even changes over time in the same patient, so patients require regular monitoring to ensure that they are not over- or under-anticoagulated. The standard measure of anticoagulation is the international normalized ratio (INR). A recent study reported polymorphisms in the *VKORC1* gene that explained up to 30% of the variation in the pharmacological response to VKAs, but few clinics take such inherited determinants of the pharmacological response into account.

In a paper published in *PLoS Medicine*, Dutch authors Pieter Reitsma and colleagues aimed to estimate the contribution of C1173T polymorphisms in the *VKORC1* gene to dose requirement for the anticoagulants acenocoumarol and phenprocoumon and to bleeding risk. In this case-control study, the authors studied 110 patients who bled during VKA therapy and 220 controls free of bleeding under the same therapy. Patients in the study were being treated with anticoagulants for venous thrombosis or were receiving anticoagulants to prevent arterial thromboembolism related to their atrial fibrillation or mechanical heart valves.

What the authors found was that to achieve the same target INR, control patients with CT and TT genotypes required less phenprocoumon or acenocoumarol than control patients with the CC genotype. Also, compared with CC individuals, carriers of at least one T allele had increased bleeding risk in the phenprocoumon users but not in acenocoumarol users.

The results, although based on a small sample size of individuals, support the suggestion that bleeding risk for T carriers is higher in phenprocoumon users than in acenocoumarol users. If this suggestion is confirmed in additional studies and extended to more frequently occurring and clinically relevant nonmajor bleeding, it may imply that CT and TT carriers should be preferentially treated with acenocoumarol, despite the fact that this anticoagulant gives poorer quality of control of treatment intensity.

At present, there is no clear explanation for risk differences between the two coumarin anticoagulants, the authors noted, although it is possible that the long half-life of phenprocoumon—a 140-hour half-life versus an 11-hour half-life of acenocoumarol—is a factor.

Although this study needs to be repeated in other populations of patients, increased bleeding risk in some individuals supports the idea that genotyping for this polymorphism in the *VKORC1* gene should be further explored. It may be that, in the future, genotyping will be a possibility for patients starting on acenocoumarol and phenprocoumon treatment in order to help identify those individuals who are at highest bleeding risk and who, thus, should be monitored more intensely.

